# Artemisinin-naphthoquine combination (ARCO™) therapy for uncomplicated falciparum malaria in adults of Papua New Guinea: A preliminary report on safety and efficacy

**DOI:** 10.1186/1475-2875-8-196

**Published:** 2009-08-12

**Authors:** Francis W Hombhanje, David Linge, Adolf Saweri, Cynthia Kuanch, Robert Jones, Stephen Toraso, Jacobed Geita, Andrew Masta, Isi Kevau, Gilbert Hiawalyer, Mathias Sapuri

**Affiliations:** 1 Faculty of Health Sciences, Divine Word University, Madang, Madang Province, Papua New Guinea; 2 School of Medicine and Health Sciences, University of Papua New Guinea, Papua New Guinea; 3 St. Mary's Private Hospital, Port Moresby, Central Province, Papua New Guinea; 4 Port Moresby General Hospital, Port Moresby, Papua New Guinea; 5 Madang Modilon Hospital, Madang, Madang Province, Papua New Guinea; 6 United Nations Family and Population Agency, Port Moresby, Papua New Guinea

## Abstract

**Background:**

The use of anti-malarial drug combinations with artemisinin or with one of its derivatives is now widely recommended to overcome drug resistance in falciparum as well as vivax malaria. The fixed oral dose artemisinin-naphthoquine combination (ANQ, ARCO™) is a newer artemisinin-based combination (ACT) therapy undergoing clinical assessment. A study was undertaken to assess the safety, efficacy and tolerability of ANQ combination in areas of multi-drug resistance to generate preliminary baseline data in adult population of Papua New Guinea.

**Methods:**

The clinical assessment was an open-labeled, two-arm, randomized study comparing ANQ combination as a single dose regimen and three days regimen (10 mg/kg/day) of chloroquine plus single dose sulphadoxine-pyrimethamine (CQ+SP) for the treatment of uncomplicated falciparum malaria with 28 days follow-up in an adult population. The primary outcome measures for efficacy were day 1, 2, 3 7, 14 and 28-day cure rates. Secondary outcomes included parasite clearance time, fever clearance time, and gametocyte carriage. The main outcome measures for safety were incidences of post-treatment clinical and laboratory adverse events.

**Results:**

Between June 2005 and July 2006, 130 patients with confirmed uncomplicated *P. falciparum *were randomly assigned to receive ANQ and CQ+SP, only 100 patients (51 in ANQ group and 49 in CQ+SP group) were evaluated for clinical and parasitological outcomes. All the patients treated with ANQ and CQ+SP showed adequate clinical and parasitological response with 28 days follow-up. The cure rate for ANQ on day 1, 2, 3, 7, 14, and 28 was 47%, 86%, 92%, 94%, 94% and 94%, respectively. Recrudescence account for 6%; all were cleared on day 21. For CQ+SP treated group the cure rates were 24%, 67%, 82%, 82%, 84% and 88%, respectively. Recrudescence accounted for 10%; all were cleared on day 28 except for one patient. Both regimens were well tolerated with no serious adverse events. The proportion of gametocyte carriers was higher in CQ+SP treated group than ANQ treatment (41% versus 12%; p < 0.05).

**Conclusion:**

While these data are not themselves sufficient, it strongly suggests that the ANQ combination as a single dose administration is safe and effective for the treatment of uncomplicated *P. falciparum *malaria in the adult population of Papua New Guinea and deserves further clinical evaluation.

## Introduction

In Papua New Guinea (PNG), malaria is one of the leading causes of morbidity and mortality, particularly among children under five years of age. The most predominant of the four species are *Plasmodium falciparum *(*P. falciparum*) and *Plasmodium vivax *(*P. vivax*) [1,2]. The vast majority of malaria-related deaths are due to *P. falciparum *infections but equally important are the *P. vivax *infections which cause severe febrile illnesses but are rarely fatal. The transmission of malaria has increased over the past decade [2,3], partly as result of lack of funds for malaria control programs, massive population movement from non-malaria areas to malaria endemic areas and vise versa for employment opportunities, climate changes, and widespread multi-drug resistance to currently available anti-malarials [4-6].

The 4-aminoquinolines (chloroquine [CQ] for adults/amodiaquine for children) have been used as the first-line anti-malarial drugs for malaria treatment for many years. However, the therapeutic efficacy of the 4-aminoquinolines, particularly of chloroquine, has declined over the years [5,6] since the mid-70s [7]. In an effort to sustain the therapeutic efficacy, the PNG National Department of Health added sulphadoxine-pyrimethamine (SP) to the 4-aminoquinolines [8]. Despite the effort clinical resistance to the CQ+SP combination emerged soon after its introduction [9]. The clinical observations are support by molecular studies where many of the clinical isolates of *P. falciparum *carry multi-drug resistant genes [10-12], which meant that new anti-malarial combinations have to be sought. The artemisinin and/or its derivatives have widely been advocated for combating multi-drug resistance [13-15]. Although artemisinin-based combination therapies (ACTs) are known to be effective anti-malarials [16,17], PNG is yet to introduce ACTs into its national malaria treatment policy. In an effort to effect policy change from CQ+ SP, which is the current first-line treatment, to ACT, to replace current first-line treatment, several different combinations have recently been evaluated in PNG [18]. The ANQ combination is one of the new generations of fixed-dose ACTs, which was evaluated in this study. The combination tablet was initially developed by the Academy of Military Medical Sciences (AMMS), Beijing, China and is currently administered as a single-dose regimen. Although clinical experiences and clinical data are very limited, available pre-clinical and clinical data claim that ANQ is safe and effective in a single dose administration [19,20]. The results of this study show ANQ to be comparatively safe, tolerable and efficacious.

## Patients and methods

### Study site and patient selection

**Figure 1:**
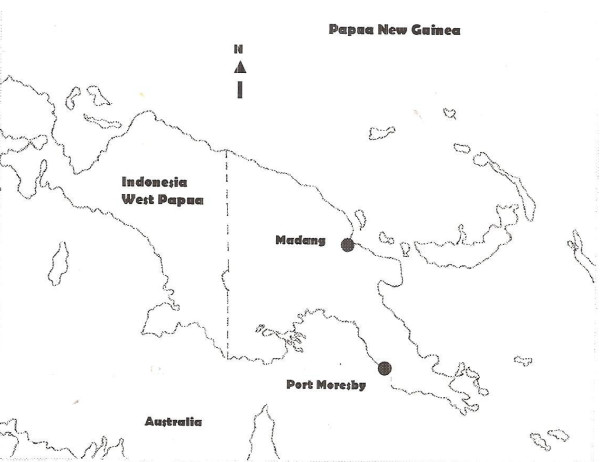
**The map of Papua New Guinea showing the locations of the study in the two regions**.

Three hospitals were selected for the study; two in Port Moresby, southern region, and Madang in the northern region (Figure 1). Moderate to high malaria transmission with the predominance of *P. falciparum *and *P. vivax *infections with high prevalence of multi-drug resistant *P. falciparum *are experienced in both regions [4-6,21]. The patients for the study were selected on the following inclusion criteria: (i) positive thick blood smear of pure *P. falciparum *infection (ii) age 14 years and over, (iii) agreed to 28-day follow-up, (iv) parasitaemia of 500 parasites/μL and over (later it was changed to ≥ 250 parasites/μL due to slow in recruiting patients), and (v) signed informed consent form. The exclusion criteria were: (i) danger signs of severe malaria, (ii) pregnancy, (iii) history of allergy to study drugs, (iv) taken anti-malarial drugs in the last seven days, and (v) medical history or concurrent systemic medical conditions. The exiting criteria from the study were: (i) completion of the 28-day period, (ii) identification of mixed species infection in the course of study, (iii) development of serious drug related toxicity, (iv) progression of signs and symptoms indicative of impending severity of malaria, and (v) developed non-malaria infections during the course of the study. The study was designed as an open-label, two-arm, randomized study comparing a single dose ANQ (ARCO™ 125/50, Kunming Pharmaceuticals, Kunming (KPC), China) with three days CQ and single dose SP (CQ+SP), the current first line treatment for malaria in PNG. The Research Committee of the School of Medicine and Health Sciences, University of Papua New Guinea, and the Medical Research Advisory Committee (MRAC) of the PNG National Department of Health (MRAC #:1529) gave the ethical approval for the study.

### Study and laboratory procedures

The trial patients were admitted to the hospital for the first three days (72-hrs). On admission (day 0), a medical history and a full physical examination were performed on all patients. Venous blood (~10 ml) was collected for biochemistry, haematology, and renal function tests. Blood smears for parasite microscopy were done on 0, 8, 16, 24, 32, 40, 48, 60 and 72 hrs before patients were discharged from hospital. Thereafter patients were asked to return to the hospital on days 7, 14, 21, and 28. On each visit a full physical examination, blood smears and collection of venous blood (5 ml) were done for each patient; blood smears for parasite microscopy and venous blood for biochemistry, haematology, and renal function tests. In addition, a questionnaire on adverse events was completed for each patient. Any patients complaining of fever or symptoms consistent with malaria, a blood smear was taken and microscopically examined during the follow-up period.

The malaria parasite counts were determined on 10% Giemsa stained thick blood smears under high power magnification (10 × 100). Parasitaemia was measured by counting a number of asexual parasites against 200 white blood cells (WBC). However, where parasites decreased following treatment such that parasites may fall below 10 parasites per 200 white blood cells, count was done against 500 WBC. The parasitaemia (parasites/μl of blood) was calculated based on the number of asexual forms seen in 200 WBC multiplied by 8000 [22]. The blood smears were pronounced negative after having examined 100 fields of a thick smear. Presence or absence of gametocytes was noted per 500 WBC with Giemsa stained thick smears. The blood smears were read by two laboratory technicians experienced (15+ years) in malaria diagnosis.

### Drug regimens

The trial patients were randomized to either of the two treatments using a table of random numbers adopted from *Geigy Scientific Tables *[23]. Patients allocated to ANQ received a total of 1000 mg of artemisinin and 400 mg naphthoquine in 8 tablets as a single dose dispensed with water. Each tablet contained 125 mg of artemisinin and 50 mg of naphthoquine. Patient allocated to CQ+SP group received CQ and SP separately. Each tablet of CQ contained 150 mg and the number of tablets given according to body weight; dispensed as 10 mg/kg daily for three days with water. Each tablet of SP contained sulphadoxine 500 mg and pyrimethamine 25 mg. SP was dispensed as a fixed dose combination tablet according to weight based on 25 mg/kg of sulphadoxine component. The maximum dosage for patients weighing more than 50 kg was four tablets per dose and patients weighing 50 kg or less received three tablets per dose. SP was given on the first day of treatment along with first dose of CQ. All the drugs were administered under close supervision of the hospital staff. None of the patients vomited within 20 minutes of taking medication, which could have necessitated repeating the same drug dosages. Medications were given in an empty stomach or two hrs after a meal. The guardians were advised to cool patients by tepid sponging if axillary temperature was ≥ 38.5°C, and paracetamol antipyretic, if deemed necessary.

### Study outcomes

The primary outcome measured for efficacy was cure rates (the proportion of cases of true treatment success judged by two negative blood smears on two consecutive occasions without recrudescence parasitaemia) on day 1, 2, 3, 7, 14, and 28. Recrudescence was defined as reappearance of peripheral parasitaemia after two or more consecutive negative blood smears. The secondary measures were, parasite clearance time (PCT) defined as time taken from the first dose of the medication to the first of two successive negative blood smears; fever clearance times (FCT_A _& FCT_B_), FCT_A _defined as time taken for the temperature to fall for the first time below 37.5°C from first dose of the medication; FCT_B _defined as time taken to become and remain afebrile during the period of the study; and proportion of patients who had gametocytes post-treatment.

### Assessment of adverse events

Interactive approach was used for monitoring adverse events. A clinician or a nurse recorded any event occurring in the trial patients during hospital sat and during follow-up. The patients were asked how they felt, if they had experienced any new "feeling" after start of treatment. Each patient was approached systematically for the presence or if they had experienced any anorexia, nausea, vomiting, abdominal pain, headache, dizziness, or any other events. Laboratory evaluation consisted of blood glucose level, liver function tests, renal function tests, and standard haematological indices at enrollment and at day 3, 7, 14, 21, and 28.

The results of the clinical and laboratory assessment were considered adverse events if they were new or increased in severity with respective to the baseline data. Adverse events were defined as any symptoms or signs that were not present on admission into the study and that were developed after the start of treatment. All adverse events, including those related to malaria were recorded and compared among treatment groups.

### Data management and analysis

The arithmetic mean and standard deviation (mean ± sd) were calculated for each patient's parasitaemia. Mean parasitaemia values were converted to percentage, baseline parasitaemia being 100%. Using Excel database and SPSS software the percentages of mean parasitaemia were plotted against time to assess the response pattern. Student's *t*-test was used in the study for statistical significance and *P *values less than 0.05 were considered to be statistically significant. Analysis of treatment outcomes was performed on the intention-to-treat basis.

## Results

One hundred and thirty (n = 130) patients were recruited into the study with positive blood smears for malaria parasites between June 2005 and July 2006. Thirty (n = 30) patients were excluded for the following reasons: 10 patients had *P. vivax*, five patients excluded for violation of the randomization order by one physician, one patient had a history of psychiatric illness, one patient had renal impairment, one patient was found be pregnant, two patients absconded after admission before treatment commenced. One hundred (n = 100) patients fulfilled the criteria for final analysis; fifty-one (n = 51) patients in ANQ group and forty-nine (n = 49) in the CQ+SP group. Adherence to the protocol was generally good: around 94% (n = 94/100) completed follow-up; 6% (n = 6) patients, two (n = 2) patients failed follow-up investigation on day 21 in ANQ group and four (n = 4) failed follow-up investigations on 28 in the CQ+SP group.

### Clinical and laboratory findings

The baseline characteristics were similar in both treatment groups (Table 1). The patients presented with varied symptoms suggestive of malaria including fever. These symptoms disappeared within seven days post-treatment in both treatment groups. None of the patients developed severe or suffered any complications of malaria during the course of the study. Laboratory data (Table 2) did not show much difference between the two treatments. Pre-treatment anaemia (Hb <10.0 g/dl) however, was observed in 10 subjects (4/51 in ANQ and 6/49 in CQ+SP), which improved upon treatment except in two patients (one in each treatment group).

**Table 1:** The pre-treatment clinical, parasitological, and demographic data of trial patients allocated ANQ and CQ+SP treatments

**Pre-treatment**	**Treatment Groups**	
Total number recruited: 130	ANQ	CQ + SP

Numbers analyzed	51	49

Male/female	27/24	25/24

Age: median (range)	27.5 (14–60)	23.0 (14–60)

Weight (Kg): median (range)	60 (43.3–98)	56.6 (42–79)

Axillary temperature (°C)	38.2 ± 1.2	38.2 ± 1.3

Mean parasitaemia* ± sd	22228.1 ± 34567.2	19761.9 ± 36888.5

* Arithmetic mean

**Table 2:** Laboratory monitoring of biological events of ANQ and CQ+SP treatments

Laboratory parameters	Treatments	D0	D3	D7	D14	21	28
**Biochemistry**							
Glucose *mm/L*	ANQ	4.9 ± 0.9	5.5 ± 1.5	4.8 ± 0.8	4.5 ± 0.8	4.5 ± 0.5	5.0 ± 1.6
	CQ+SP	5.0 ± 1.0	5.5 ± 1.5	4.9 ± 0.8	4.5 ± 0.8	4.6 ± 0.5	5.2 ± 1.6

**Liver function**							
*AST *Units/L*	ANQ	43.2 ± 16.4	43.7 ± 22.6	41.6 ± 32.1	35.7 ± 17.9	31.0 ± 10.4	33.4 ± 10.2
	CQ+SP	44.2 ± 16.4	43.2 ± 16.4	42.2 ± 32.1	35.2 ± 17.9	31.0 ± 10.4	35.4 ± 10.2

**ALT *Units/L*	ANQ	44.1 ± 20.9	47.9 ± 24.9	53.0 ± 49.2	57.3 ± 33.7	36.4 ± 14.8	36.3 ± 21.4
	CQ+SP	46.1 ± 20.9	47.9 ± 24.9	54.0 ± 49.2	57.3 ± 34.0	36.4 ± 14.9	35.3 ± 21.4

Bilirubin *μm/L*	ANQ	19.4 ± 14.3	12.1 ± 8.8	10.7 ± 5.0	8.3 ± 1.5	8.8 ± 1.3	8.8 ± 1.9
	CQ+SP	18.4 ± 14.3	12.1 ± 8.8	10.6 ± 5.0	8.3 ± 1.5	8.0 ± 1.3	8.8 ± 2.0

Alkaline phosphatase *Units/L*	ANQ	75.9 ± 18.6	81.8 ± 24.7	87.5 ± 27.5	91.3 ± 29.5	80.5 ± 24.3	95.1 ± 38.1
	CQ+SP	76.4 ± 20.2	83.4 ± 23.1	86.1 ± 25.2	91.8 ± 28.4	77.6 ± 21.5	84.0 ± 39.0

#GGT *Units/L*	ANQ	39.5 ± 22.7	40.3 ± 24.8	34.7 ± 22.3	29.5 ± 13.5	16.8 ± 11.9	22.5 ± 7.4
	CQ+SP	40.5 ± 22.7	40.3 ± 24.8	34.7 ± 22.3	29.5 ± 13.5	16.3 ± 12.9	22.5 ± 7.5

**Renal function**							
Creatinine *μm/L*	ANQ	92.7 ± 19.4	91.8 ± 14.2	91.9 ± 18.1	87.9 ± 13.7	85.9 ± 10.9	79.3 ± 17.5
	CQ+SP	93.3 ± 19.3	92.8 ± 14.4	93.5 ± 19.1	88.9 ± 14.0	85.3 ± 11.5	80.4 ± 18.4

**Haematology**							
Haemoglobin *g/dl*	ANQ	10.8 ± 2.3	10.6 ± 1.8	10.4 ± 1.2	10.7 ± 1.0	11.1 ± 1.7	11.6 ± 1.4
	CQ+SP	11.0 ± 2.3	10.7 ± 1.9	10.5 ± 1.3	11.0 ± 0.9	11.1 ± 1.6	11.6 ± 1.5

WBC *×10*^*9*^/*L*	ANQ	5.2 ± 2.4	5.4 ± 1.7	5.5 ± 1.7	6.5 ± 2.5	6.6 ± 1.6	6.1 ± 1.7
	CQ+SP	5.4 ± 2.3	5.4 ± 1.6	5.8 ± 1.6	6.5 ± 2.3	6.6 ± 1.7	6.2 ± 1.8

**Electrolytes**							
Sodium *mm/L*	ANQ	134.9 ± 5.6	138.2 ± 3.4	135.6 ± 7.6	137.2 ± 2.6	136.5 ± 3.1	139.6 ± 5.4
	CQ+SP	135.9 ± 5.5	138.3 ± 3.3	136.1 ± 7.1	137.2 ± 2.4	136.4 ± 2.9	140.0 ± 5.5

Potassium *mm/L*	ANQ	3.8 ± 0.7	4.1 ± 1.1	4.2 ± 0.8	4.1 ± 0.7	3.9 ± 0.3	4.0 ± 0.7
	CQ+SP	3.8 ± 0.7	4.1 ± 0.7	4.2 ± 0.7	4.0 ± 0.7	4.0 ± 0.7	4.1 ± 1.0

*AST = Aspartate aminotransferase ** ALT = Alanine aminotransferase #GGT = Gamma-glutamyltransferase

#### Fever clearance

**Figure 2:**
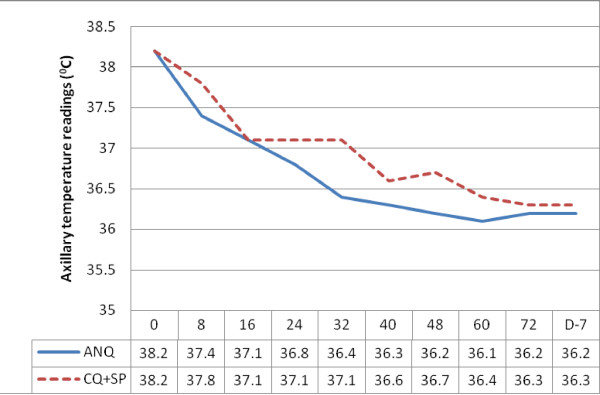
**The response of the patients' axillary body temperatures (°C = degree Celsius) after the start of the two treatments (ANQ versus CQ+SP)**.

Only 76 patients of 100 patients were evaluated for FCT; remaining 24 patients had a temperature <37.5°C on admission. However, within 24 hrs, 50% of the afebrile patients in each group developed fever (≥37.5°C). The general profile of temperature resolution in both treatment groups is shown in Figure 2. The mean FCT_A _was 13.6 ± 9.6 hr in ANQ group versus 17.2 ± 9.9 hr in CQ+SP (*p *> 0.05). The mean FCT_B _was 19.8 ± 12.7 hr in ANQ group versus 31.7 ± 21.5 hr in CQ+SP group (*p *> 0.05).

#### Parasitological responses

**Figure 3:**
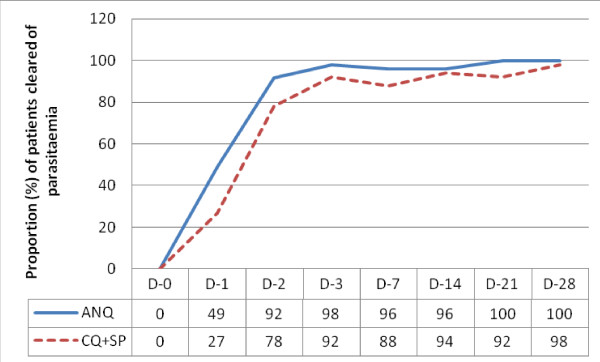
**The cumulative data of proportion (%) of patients cleared of parasitaemia with time**.

The cure rates for ANQ on day 1, 2, 3, 7, 14, and 28 were 47%, 86%, 92%, 94%, 94% and 94%, respectively. Recrudescence account for 6% (n = 3/51) and the recrudescence parasitaemia cleared in all on day 21. For CQ+SP treated group, the cure rates were 24%, 67%, 82%, 82%, 84% and 88%, respectively. Recrudescence accounted for 10% (n = 5/49) and the recrudescence parasitaemia cleared in all on day 28. One patient (1/49; 2%) in CQ+SP group remained parasitaemic throughout the study period. Figure 3 shows cumulative data of proportion (%) of patients cleared of parasitaemia with time.

**Figure 4:**
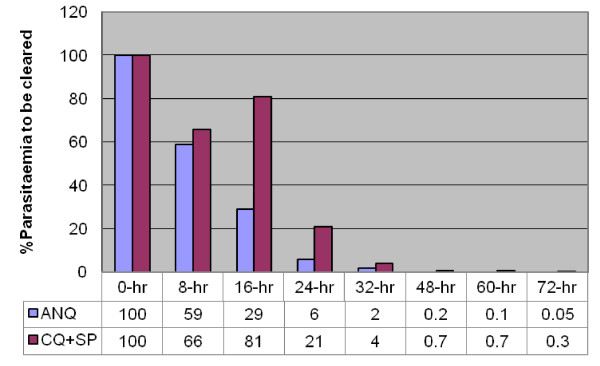
**The clearance of blood parasitaemia in the first seventy-two hours (72 hrs) after the start of the two treatments (ANQ versus CQ+SP)**.

The parasite clearance profile for the first 72 hrs is shown in Figure 4. The ANQ treatment was associated with rapid reduction in parasites density within the initial 24 hr of treatment. Unfortunately, similar response was not observed with CQ+SP treatment. A rise in post-treatment parasite density was observed at 16 hr after the start of CQ+SP treatment. The reason for this phenomenon is unknown but has been observed previously [24,25]. The median parasite clearance time (PCT) for CQ+SP was 40 hrs (range: 16–72 hr) versus 24 hrs (range: 16–72 hr) for ANQ.

### Gametocyte carriers

Pre-treatment gametocytaemia was observed in 6 patients (n = 3/51 in ANQ group; n = 3/49 in CQ+SP group). After the start of the two treatments, 12% (n = 6/51) of the patients in ANQ treated group developed gametocytes versus 41% (n = 20/49) in CQ+SP group (p < 0.05). The post-treatment gametocyte density was not evaluated as this was not part of the study design.

### Adverse Events Reported

Both treatment regimens were well tolerated with no serious adverse events being reported or detected. Post-treatment emergent symptoms that worth noting were transient deafness (5%, n = 5/100; 4 in ANQ group; one in CQ+SP group), itchiness (3%, n = 3/100; one in ANQ group, two in CQ+SP group), skin rash (2% n = 2/100; one for each treatment group), and dark urine (1%, n = 1/100, ANQ group only). The rate of vomiting after initiating treatment was low (one in the ANQ group, two in the CQ+SP group) and did not differ greatly between the two treatment groups. Several patients reported treatment emergent symptoms such as anorexia, nausea, dizziness, abdominal pain and headache within 24-hrs of admission. However, it was difficult to discern which of the symptoms was malaria related and which were due to drug treatment. One of the patients developed psychosis ~5 hours after oral administration of ANQ. The patient had low plasma glucose (2.0 mmol/L; normal range: 3.0 – 5.0 mmol/L), which was corrected with dextrose saline. The patient underwent psychiatric assessment (by a psychiatrist) and organic causes were excluded. The patient's condition during post-treatment follow-up was uneventful. Hypoglycaemia rather than ANQ medication could have been responsible for the psychosis.

## Discussion

Both treatments in this study cleared parasitaemia adequately and reliably. However, ANQ had the more rapid anti-malarial action, resulting in almost 50% of the trial patients being cleared of parasites in the first 24 hours. Moreover, ANQ treatment significantly suppressed gametocytogenesis than the CQ+SP treatment. What makes this ACT unique is the claim that it is effective when administered as a single dose, whereas other ACTs require multiple dosing [26,27]. The results of this study support this claim and further affirm the highly potent anti-malarial and anti-gametocytocidal properties of artemisinin-based combinations [15,28].

The ANQ combination possesses benefits of both short-acting artemisinin, and long-acting naphthoquine combined into one tablet. The administration of 8 tablets in a single dose seems an optimal dosage for the trialed population in treating uncomplicated falciparum malaria infections. The dosage is safe and effective in clearing the blood parasitaemia. Rapid reduction in blood parasitaemia by ANQ, without post-treatment surge in parasitaemia, is critical for preventing potential complications or progression to severe and complicated malaria. However, whether rapid clearance of parasites by ANQ makes ANQ a more effective drug combination than CQ+SP is to be ascertained with further studies. Interestingly, CQ+SP combination still remain relatively effective at dosage given (30 mg/kg over three days), despite reports of seemingly moderate to high levels of multi-drug resistance in the study areas [4-6,21]. However, increased CQ+SP post-treatment gametocytogenesis observed in this study and also in other studies [29-31] is a public health concern. Gametocytes involved in "post-treatment transmission" are more likely to carry and spread drug-resistant genetic alleles [32]. The ACTs including ANQ combination therapy provide better anti-gametocyte activities; a potential public health benefit that could further be explored. Recent studies show that ACTs that lack either 4-aminoquinolines or SP in combination therapies provided better anti-gametocytocidal activity than those combinations that included 4-aminoquinolines or SP [33].

One of the key elements in any drug development and evaluation is the issue of safety of the population for which the drug is intended. Data from this study indicate that the use of ANQ combination is relatively safe, both from the clinical and laboratory perspectives. However, the quinine-like transient deafness reported by several patients who received ANQ combination requires further investigation. Although the sample size in this study is insufficient, larger studies are needed to define the safety and efficacy in different populations including children and pregnant women and in different ethnic settings. This ACT is uniquely novel in that it can be administered less frequently (as a single one day dose or twice a day dose) and yet produces almost equivalent cure rates to more frequently administered drug regimens such as CQ+SP combination in this study or artemether-lumefantrine combination in other studies [34]. Hence, the ANQ combination therapy may be considered where compliance or adherence to treatment is a serious issue.

In conclusion, although both combinations assessed in this study provided relatively equivalent therapeutic responses (efficacy), tolerability, and safety profiles at day 28, the anti-malarial action of ANQ was more rapid than CQ+SP. The ANQ combination is a potential new generation ACT, which deserves further clinical evaluation.

## Competing interests

The authors declare that they have no competing interests.

## Authors' contributions

FWH designed the study protocol, supervised the study and prepared the manuscript. The design of the study protocol was assisted by DL, AS, AM, who were also involved in the conduct of the study. RJ and CK were responsible for the conducted of the study in Port Moresby while ST and JG were responsible for the conduct of the study in Madang. IK and MS were independent clinical assessors while GH was appointed as an independent clinical monitor and provided logistical support to the study. All the authors agreed to the content of the text.
